# Supplementary material for: Multi-omic phenotyping of MAPT V337M neurons reveals early changes in axonogenesis and tau phosphorylation
Source: NPJ Dement. 2026 Apr 25;2(1):24. doi: 10.1038/s44400-026-00076-w (PMC13110130; doi:10.1038/s44400-026-00076-w)
Supplement: Supplementary file 1 — 44400_2026_76_MOESM1_ESM [file 44400_2026_76_MOESM1_ESM.pdf]

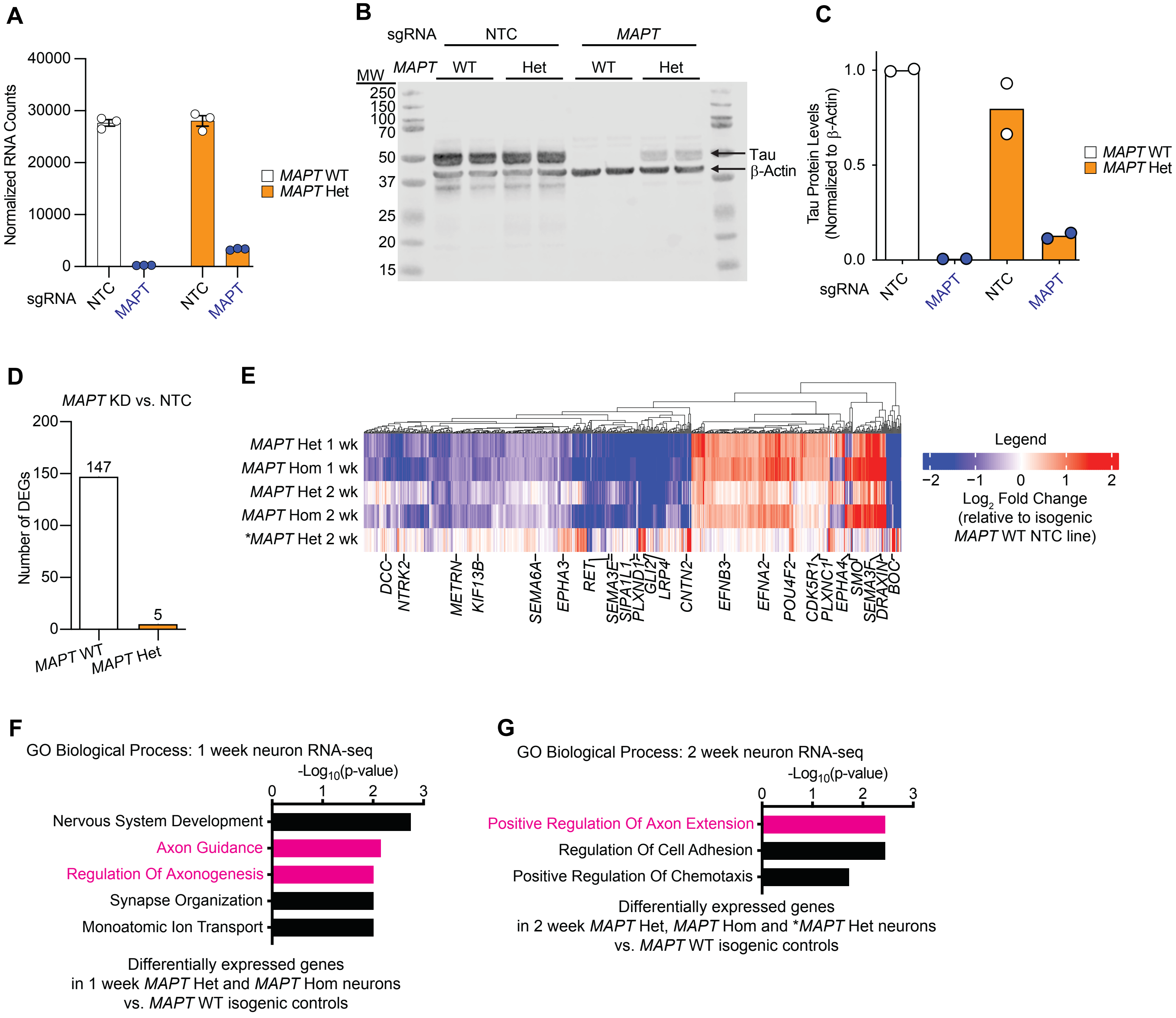

Figure S1: The *MAPT* V337M mutation and *MAPT* knockdown perturb gene expression of axonogenesis-related genes. (A) Normalized RNA counts of *MAPT* from the RNA-seq experiment described in Figure 1B showing tau knockdown in *MAPT* WT and *MAPT* Het neurons. **(B)** Western blot measuring tau knockdown in MAPT WT and MAPT Het neurons. Two replicates (individual wells) of neurons were harvested after two weeks of differentiation. **(C)** Quantification of the western blot in (B). **(D)** Bar plot showing the number of differentially expressed genes due to *MAPT* KD in either *MAPT* WT or *MAPT* Het neurons. **(E)** Heatmap of RNA-seq from *MAPT* Het, *MAPT* Hom and **MAPT* Het neurons vs. isogenic controls at 1 week or 2 weeks of differentiation. Differentially expressed genes related to axon guidance or axonogenesis are labeled. **(F)** GO term enrichment analysis of one-week neurons from the RNA-seq experiment in (A). Genes that are differentially expressed in both *MAPT* Het and *MAPT* Hom vs. *MAPT* WT were analyzed with Enrichr, and top terms were plotted. Pathways related to axonogenesis and neuron morphology are colored magenta. **(G)** GO term enrichment analysis of two-week old neurons from the RNA-seq experiment in (A). Genes that are differentially expressed in both *MAPT* Het, *MAPT* Hom and **MAPT* Het vs. their isogenic *MAPT* WT controls were analyzed with Enrichr, and top terms were plotted. Pathways related to axonogenesis and neuron morphology are colored magenta.

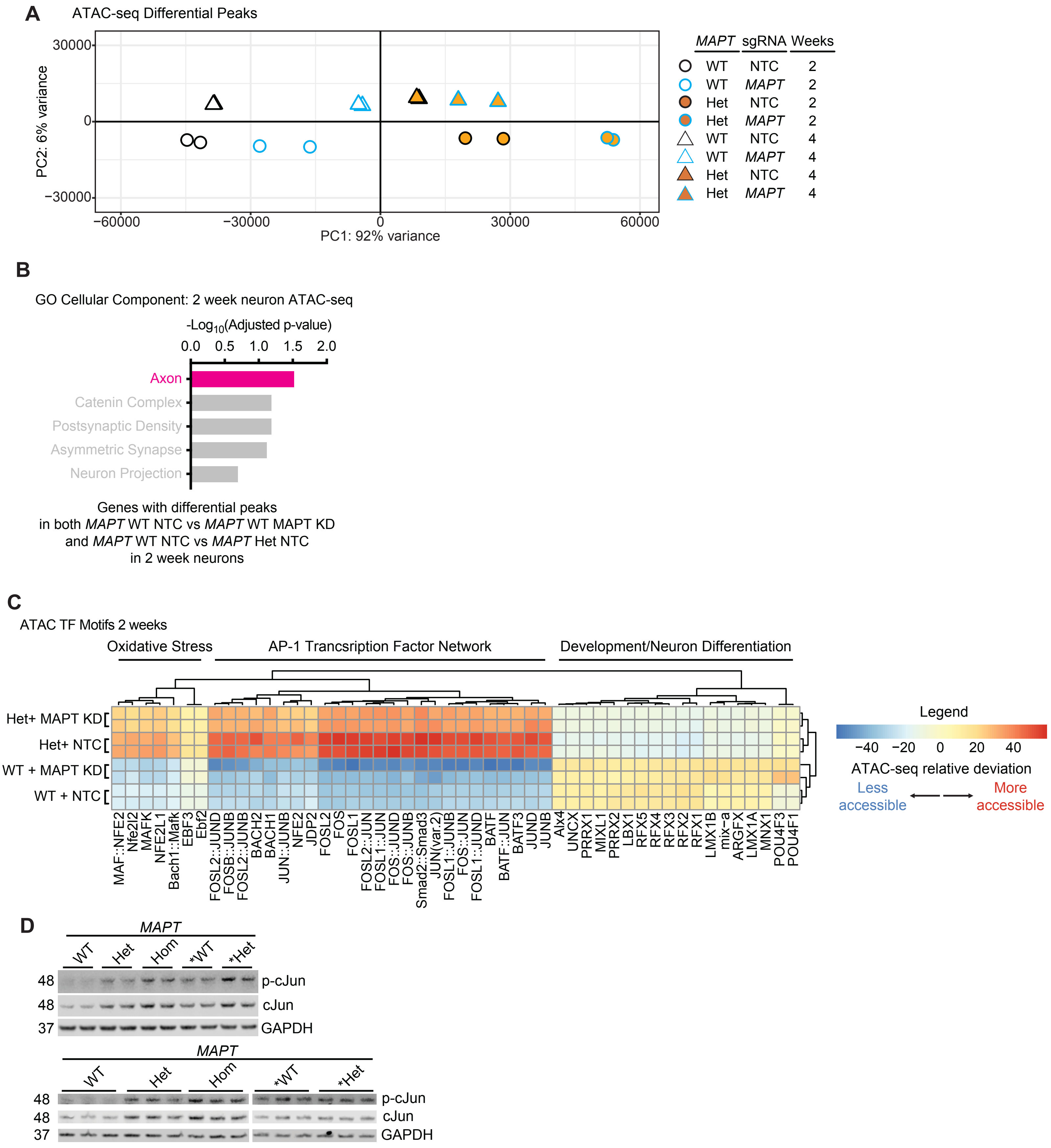

Figure S2: The *MAPT* V337M mutation and *MAPT* knockdown perturb chromatin accessibility of AP-1 transcription factor network motifs. (A) PCA plot of ATAC-seq differential peaks at 2 and 4 weeks of differentiation. Two replicates (individual wells) of neurons were harvested at each timepoint. **(B)** GO term enrichment analysis using Cellular Component on genes in 2-week neurons with differential ATAC-seq peaks in both *MAPT* WT *MAPT* KD and *MAPT* Het NTC vs. *MAPT* WT NTC. The top five terms were plotted, and non-significant terms are labeled in grey. *(continued on next page)*

(C) Heatmap showing the relative deviation of transcription factor motifs with significantly different accessibility in *MAPT* WT and *MAPT* Het neurons +/- tau knockdown. Two replicates (individual wells) of neurons were harvested at two weeks of differentiation. GO term enrichment analysis was used on clusters of transcription factors to categorize clusters. **(D)** Western blots for cJun and p-cJun in neurons at one week of differentiation. For each genotype, two independent differentiations with a total of five wells of neurons were analyzed for cJun, p-cJun and GAPDH levels.

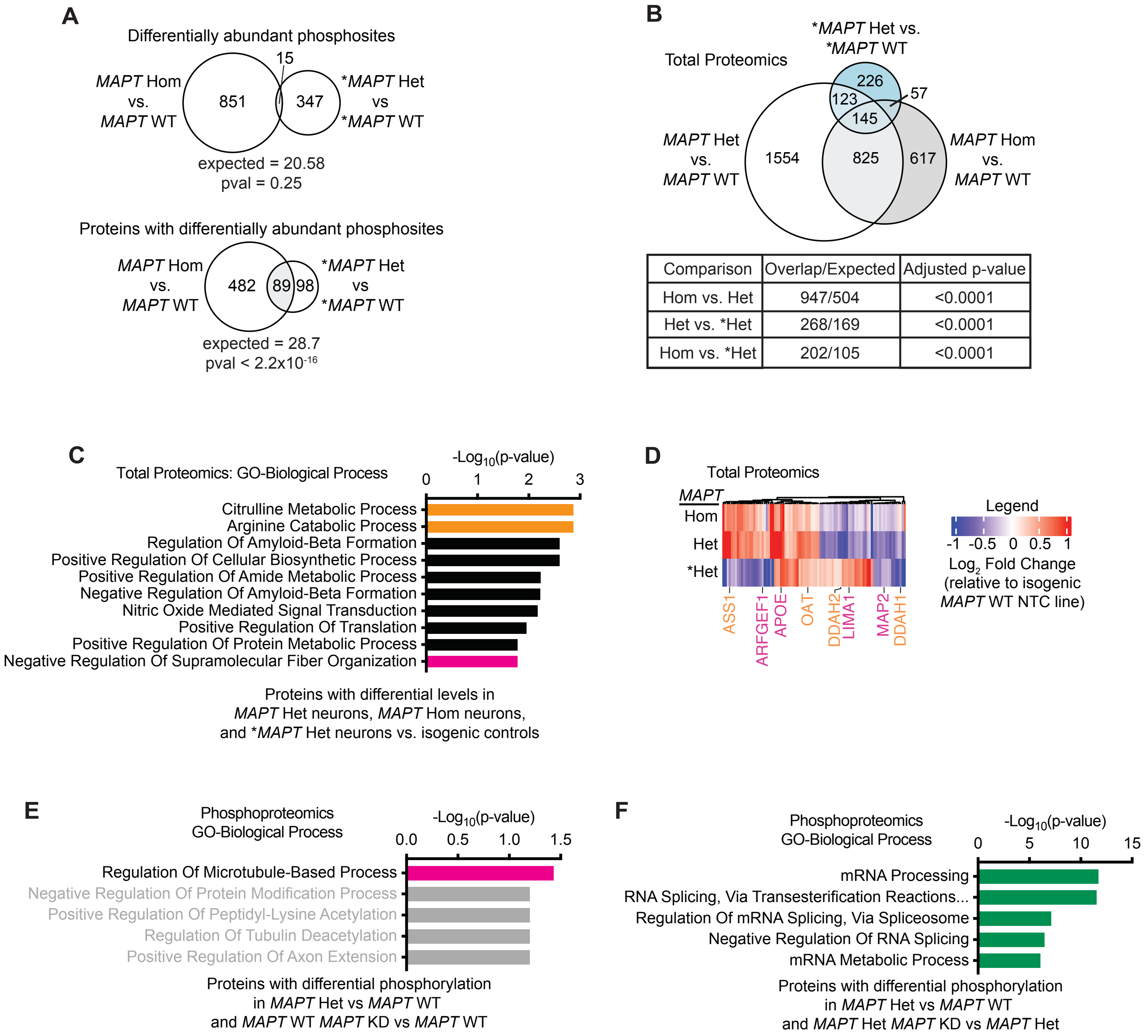

Comparison	Overlap/Expected	Adjusted p-value
Hom vs. Het	947/504	<0.0001
Het vs. *Het	268/169	<0.0001
Hom vs. *Het	202/105	<0.0001

Figure S3: The *MAPT* V337M mutation and *MAPT* knockdown cause phosphorylation changes in axonogenesis and splicing proteins. (A) (Top) Overlap between differential phosphosites in neurons derived from iPSCs edited to introduce the homozygous *MAPT* V337M mutation (*MAPT* Hom)vs. isogenic controls (*MAPT* WT). Four replicates (independent 150mm dishes) of neurons for each genotype/sgRNA combination were harvested after one week of differentiation, and the phosphoproteome was measured using mass spectrometry. Significance was calculated using Fisher's Exact Test. (Bottom) Overlap between proteins with differential phosphorylation in both datasets. Significance was calculated using Fisher's Exact Test. **(B)** Overlap between proteomic changes in *MAPT* Hom neurons, neurons derived from iPSCs edited to have the heterozygous *MAPT* V337M mutation (*MAPT* Het) and neurons derived from patient iPSCs with the heterozygous *MAPT* V337M mutation (**MAPT* Het) vs. isogenic controls (*MAPT* WT or **MAPT* WT). Four replicates (independent 150mm dishes) of neurons for each genotype/sgRNA combination were harvested after one week of differentiation, and the total proteome was measured using mass spectrometry. Significance was calculated using multiple t-tests adjusted with Šidák single-step correction. *(continued on next page)*

Significantly differential proteins in all three datasets were filtered to identify 145 conserved proteins. **(C)** GO term enrichment of the 145 proteins with differential abundance in *MAPT* Hom, *MAPT* Het and **MAPT* Het neurons compared to isogenic controls. Top non-overlapping significant terms are shown. Term names are colored to match relevant gene names in the heatmap in (C). **(D)** Heatmap showing the Log2 fold change of protein abundance for the 145 proteins with differential abundance in *MAPT* Hom, *MAPT* Het and **MAPT* Het neurons vs. isogenic *MAPT* WT neurons. Proteins within enriched GO terms are labeled and colored according to the shared pathways. **(E)** GO term analysis of phospoproteins with differential phosphorylation in *MAPT* Het NTC and *MAPT* WT *MAPT* KD vs. *MAPT* WT NTC. Non-significant terms are labeled by grey bars. Regulation of Microtubule-based process is labeled by a magenta bar due to its overlap with axon-related terms. **(F)** GO term analysis of phosphoproteins with differential phosphorylation in *MAPT* Het NTC vs. *MAPT* WT NTC and *MAPT* Het *MAPT* KD vs. *MAPT* Het NTC. Terms related to RNA processing and splicing are marked by green bars.

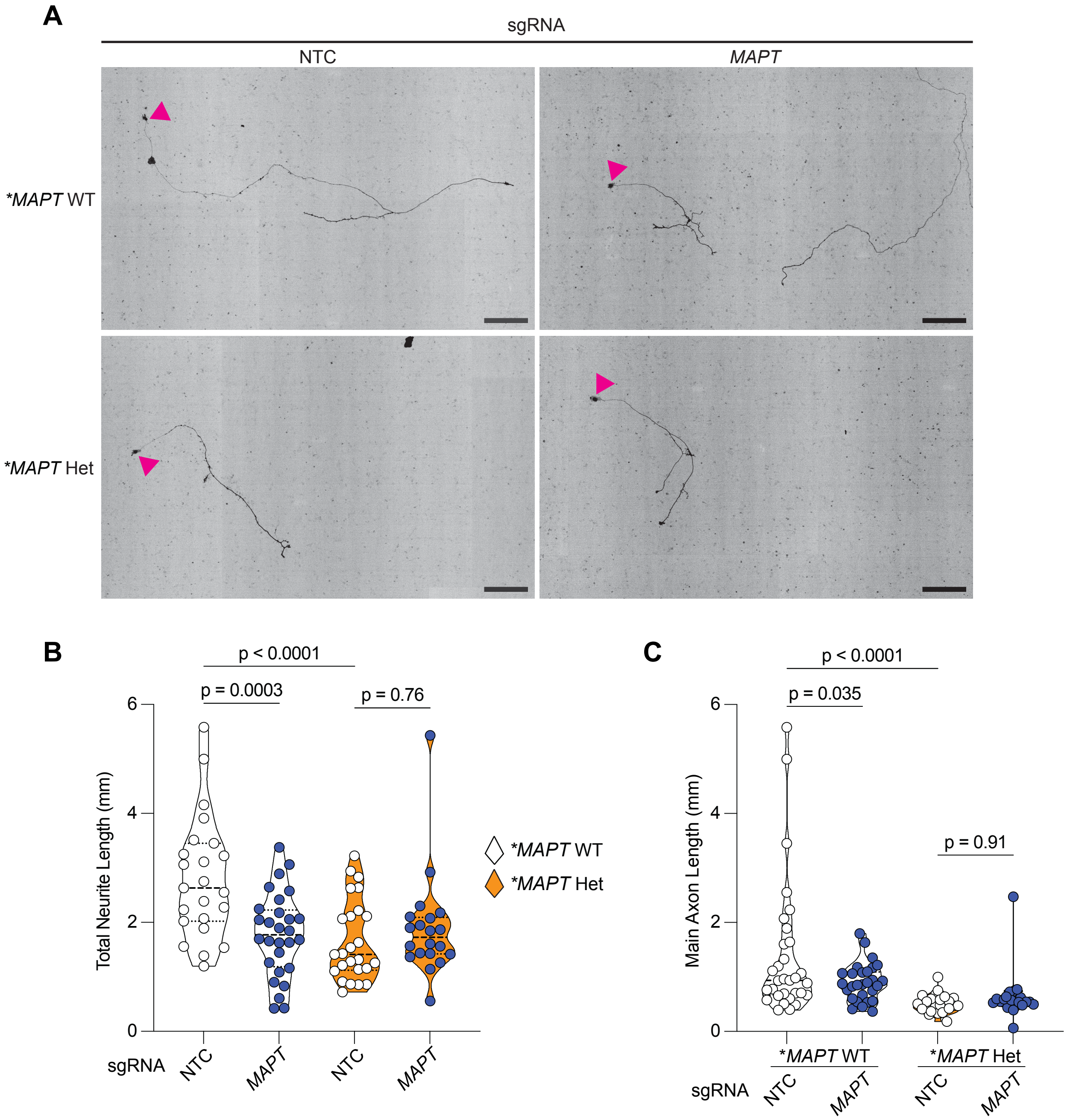

Figure S4: The *MAPT* V337M mutation and *MAPT* knockdown decrease main axon length and total neurite length in patient-derived neurons. (A) Representative images of neurons labeled with mNeonGreen. The scale bars are 200μm. Magenta arrowhead indicates the nucleus.
(B-C) Quantification of total neurite length (B) and main axon length (C). Significance was calculated using one-way ANOVA with Šidák's multiple comparison test.

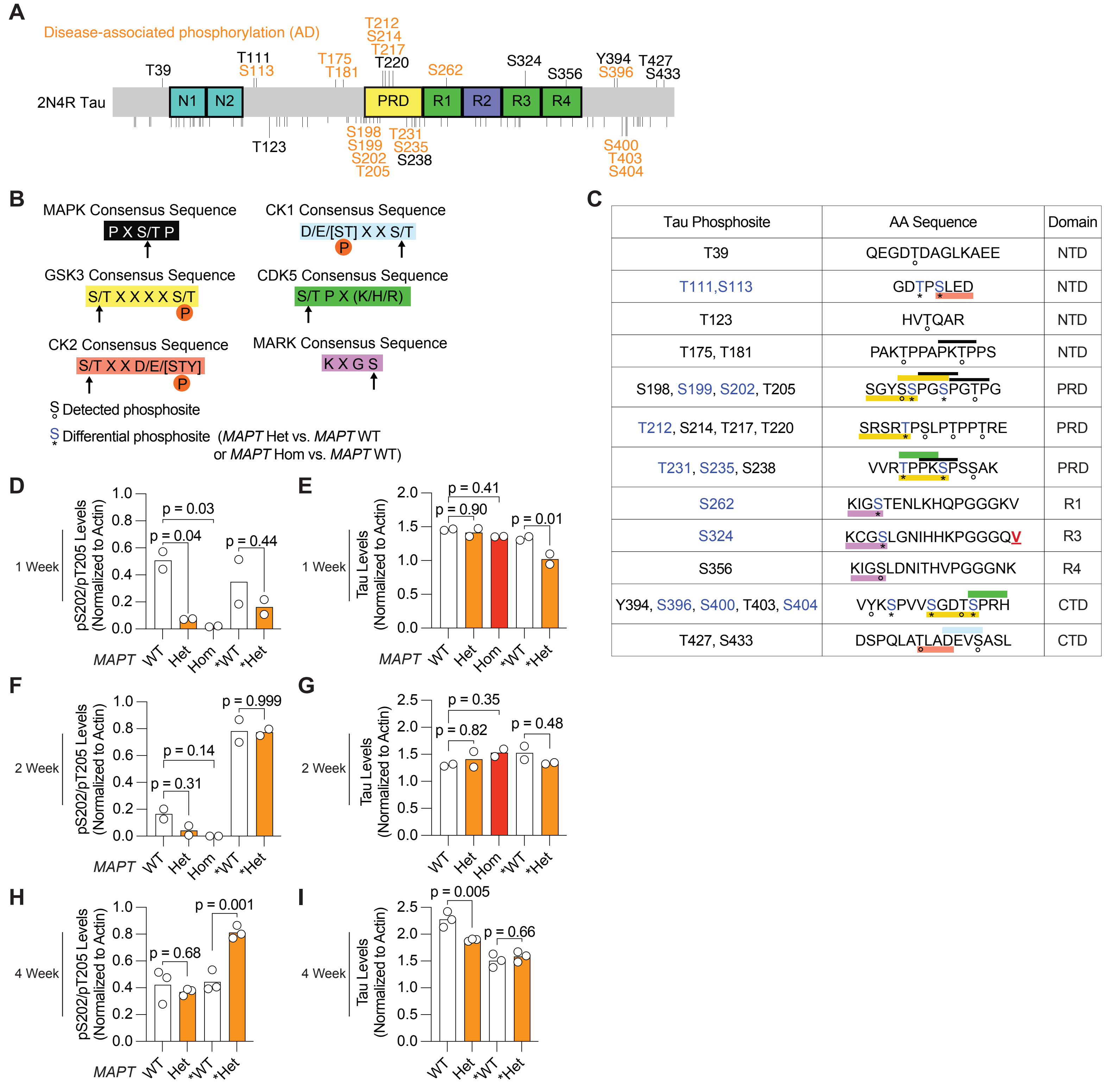

Tau Phosphosite	AA Sequence	Domain
T39	QEGDTDAGLKAEE	NTD
T111,S113	GDTPSLED	NTD
T123	HVTQAR	NTD
T175, T181	PAKTPPAPKTPPS	NTD
S198, S199, S202, T205	SGYSSPGSPGTPG	PRD
T212, S214, T217, T220	SRSRTPSLPTPPTRE	PRD
T231, S235, S238	VVRTPPKSPSSAK	PRD
S262	KIGSTENLKHQPGGGKV	R1
S324	KCGSLGNIHHKPGGGQV	R3
S356	KIGSLDNITHVPGGGNK	R4
Y394, S396, S400, T403, S404	VYKSPVVSGDTSPRH	CTD
T427, S433	DSPQLATLADEVSASL	CTD

Figure S5: Neurons with the *MAPT* V337M mutation have decreased tau phosphorylation at disease-associated phosphorylation sites. (A) Protein domain map of 2N4R tau. Phosphosites detected in this study are labeled, with disease-associated phosphorylation sites from AD labeled in orange. Phosphosites not detected in this study are marked with a small black line and are unlabeled. Domain abbreviations are as follows: N-terminal inserts (N1,N2), proline rich domain (PRD), microtubule binding repeats (R1, R2, R3, R4). **(B)** Consensus sequences for tau kinases. Kinase consensus sequences are annotated with colored boxes, with priming sites marked with a "P" in an orange circle. **(C)** Detected tau phosphosites are shown with their sequence context. Phosphorylation sites that are differential between either *MAPT* V337M heterozygous (MAPT Het) or *MAPT* V337M homozygous (*MAPT* Hom) are labeled blue with an asterisk, and detected phosphosites are labeled with an open circle. V337 is labeled with a bold/underlined red V. The domains abbreviated as follows: N-terminal projection domain (NTD), proline rich domain (PRD), Microtubule binding repeats (R1, R3, R4), C-terminal domain (CTD). **(D-I)** Quantification of western blots in Figure 3C. Significance was calculated using one-way ANOVA with Šidák's multiple comparison test.

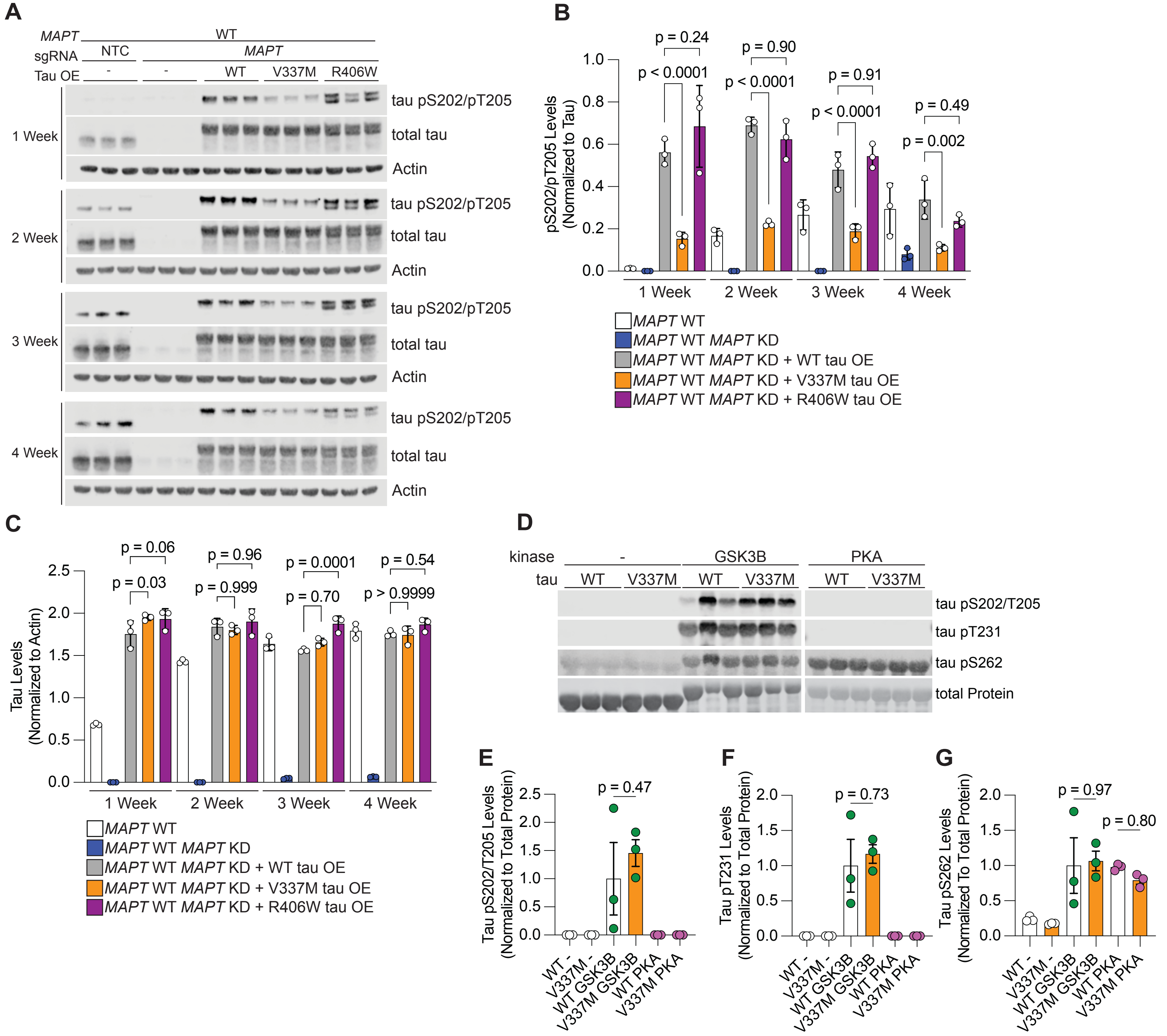

Figure S6: V337M tau is hypophosphorylated when overexpressed in *MAPT* WT *MAPT* KD neurons.
(A) Timecourse of tau pS202/pT205 and tau levels in neurons overexpressing WT, V337M or R406W tau.
(B-C) Quantification of tau pS202/pT205 levels (B) and total tau levels (C) from the blots in (A). Significance was calculated using one-way ANOVA with Šidák's multiple comparison test.
(D) Western blot measuring in vitro tau phosphorylation with GSK3B or PKA.
(E-G) Quantification of western blots in (D). Significance was calculated using one-way ANOVA with Šidák's multiple comparison test.

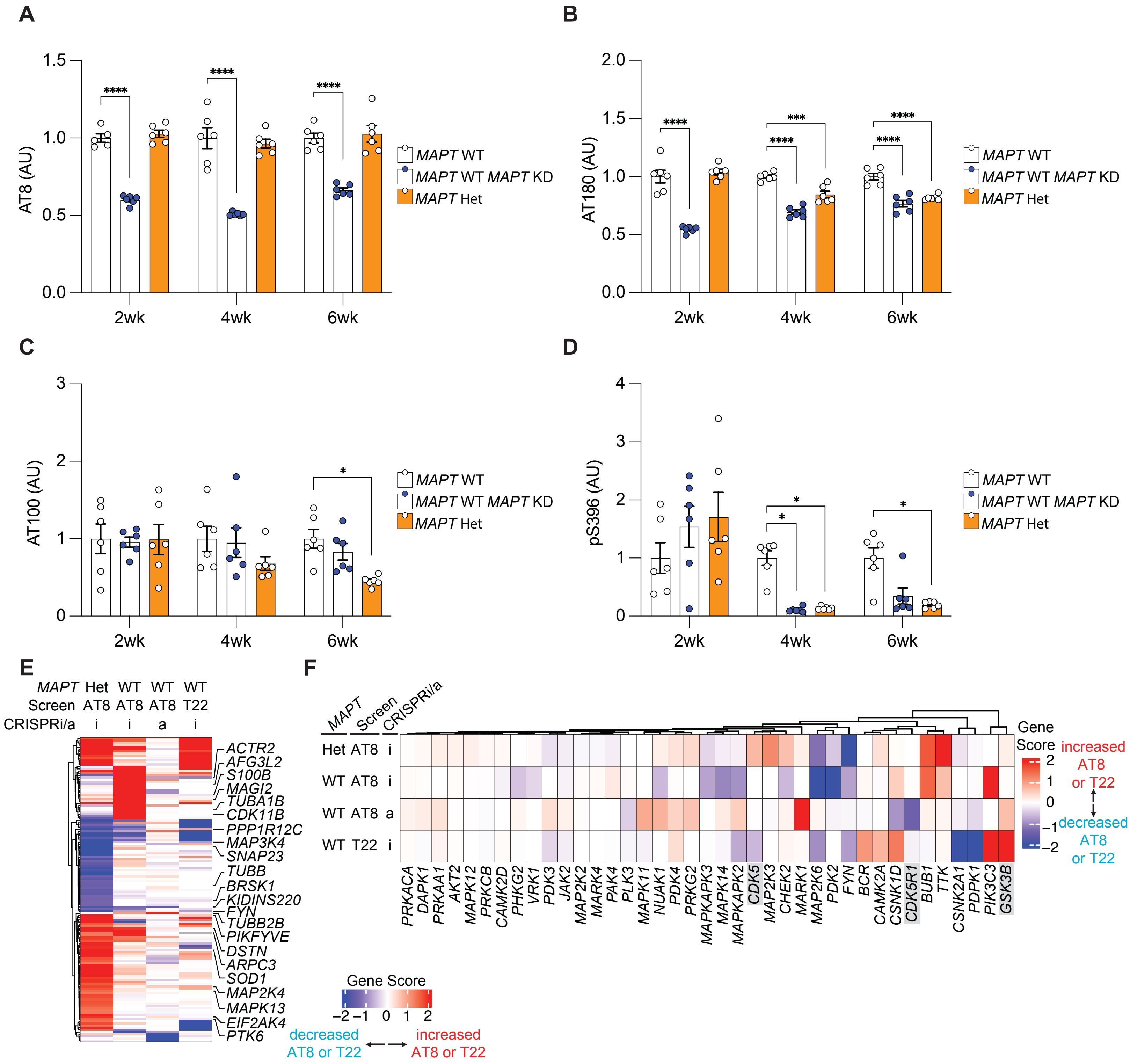

Figure S7: Functional genomics uncovers regulators of tau phosphorylation in *MAPT* WT and *MAPT* Het neurons. (A-D) Bar plots showing the median intensity of AT8 (A), AT180 (B), AT100 (C) and pS396 (D) in 2-week ***MAPT*** WT, ***MAPT*** KD and ***MAPT*** Het neurons. AT8 was selected for CRISPR screening due to high reproducibility across timepoints and AT8 detection in both ***MAPT*** WT and ***MAPT*** Het neurons at 2 weeks of differentiaton. **(E)** Heatmap of hits from the CRISPRi and CRISPRa AT8 screens and the CRISPRi T22 screen. Many of the AT8 hits from the three screens do not modify T22 levels and are therefore unlikely to be due to modifying tau levels [90]. Genes related to cytoskeleton, neuron projection development or the p38 MAPK pathway are annotated. **(F)** Heatmap of AT8 and T22 screens with the kinases predicted to have differential activity in Figure 3I. Selected kinases predicted to have differential activity in ***MAPT*** V337M neurons with particular disease relevance that did not have a phenotype in the AT8 screens are highlighted with grey boxes.

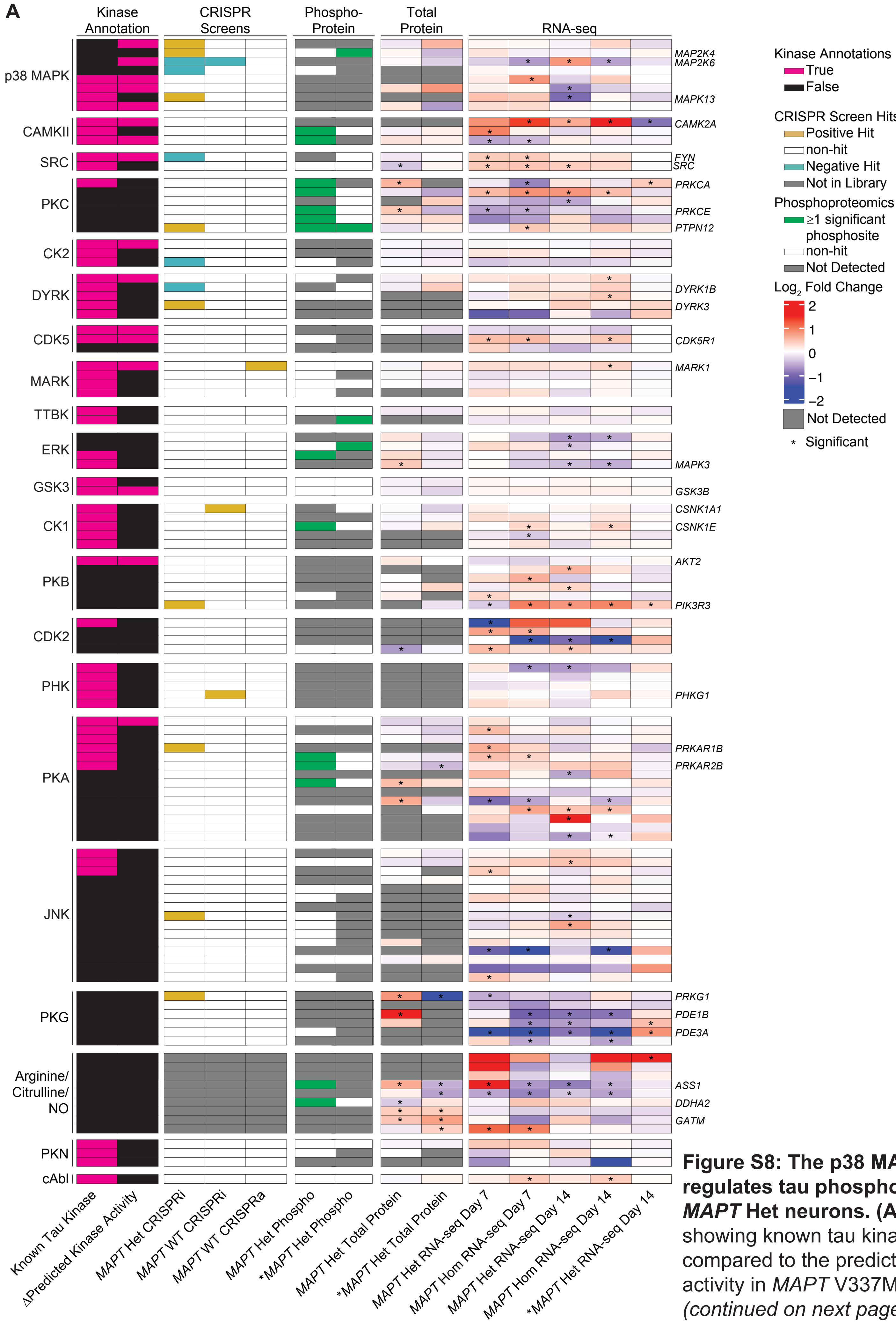

Figure S8: The p38 MAPK pathway regulates tau phosphorylation in *MAPT* Het neurons. (A) Heatmap showing known tau kinases compared to the predicted kinase activity in *MAPT* V337M neurons, *(continued on next page)*

CRISPRi/a screens, phosphoproteomics, total proteomics and transcriptomics. Several kinases in the p38 MAPK pathway modulate pS202/pT205 phosphorylation, though most were not detected in the phosphoproteomics. Most of these genes were not differentially expressed at the protein or RNA level. PKG and the Arginine/Citrulline genes were included because many of these genes were differentially expressed at the RNA and protein level as a counter example to the p38 MAPK pathway.

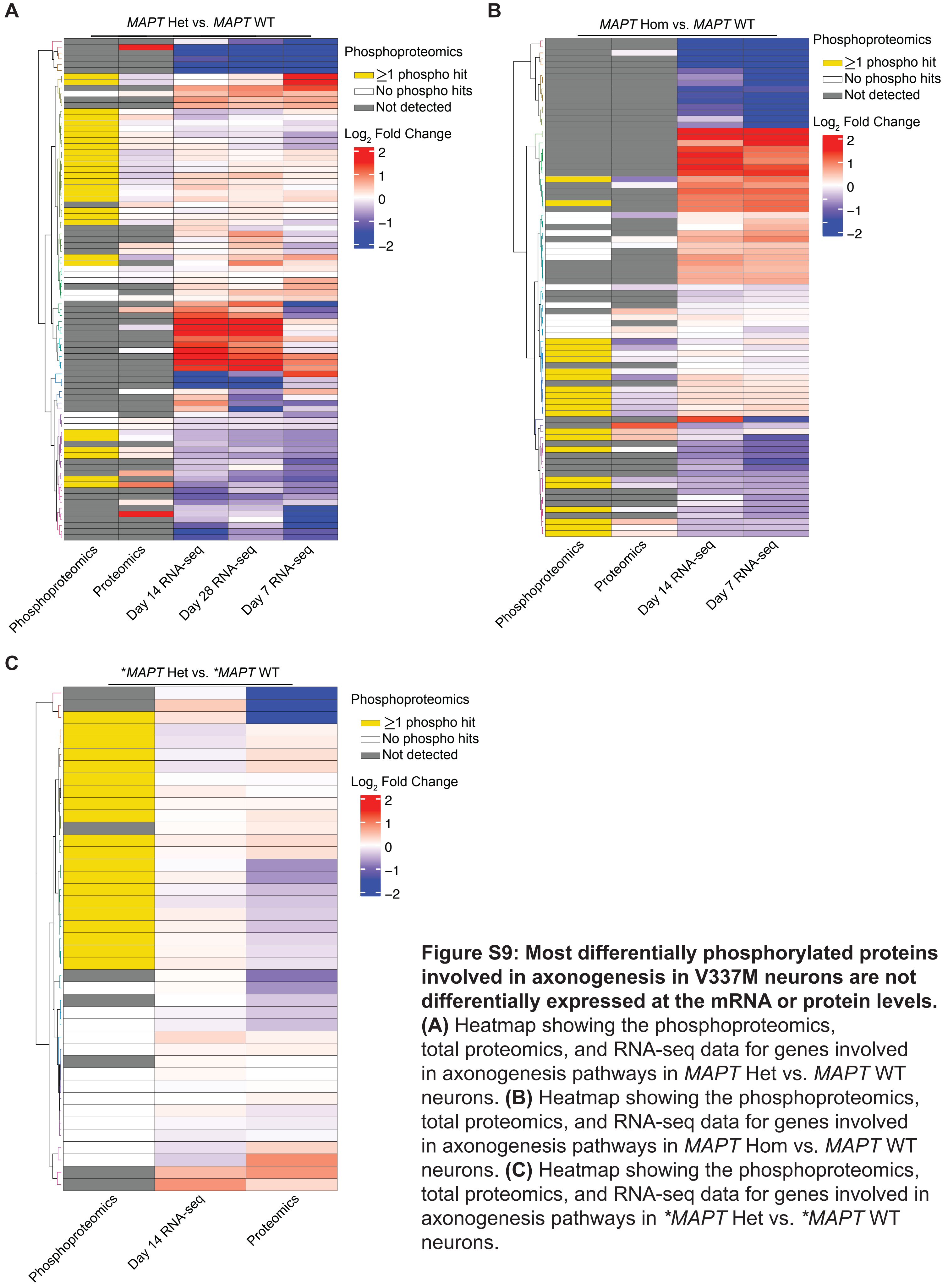

Figure S9: Most differentially phosphorylated proteins involved in axonogenesis in V337M neurons are not differentially expressed at the mRNA or protein levels. **(A)** Heatmap showing the phosphoproteomics, total proteomics, and RNA-seq data for genes involved in axonogenesis pathways in *MAPT* Het vs. *MAPT* WT neurons. **(B)** Heatmap showing the phosphoproteomics, total proteomics, and RNA-seq data for genes involved in axonogenesis pathways in *MAPT* Hom vs. *MAPT* WT neurons. **(C)** Heatmap showing the phosphoproteomics, total proteomics, and RNA-seq data for genes involved in axonogenesis pathways in **MAPT* Het vs. **MAPT* WT neurons.

Source data for Fig. S2D

cJun - 800 channel

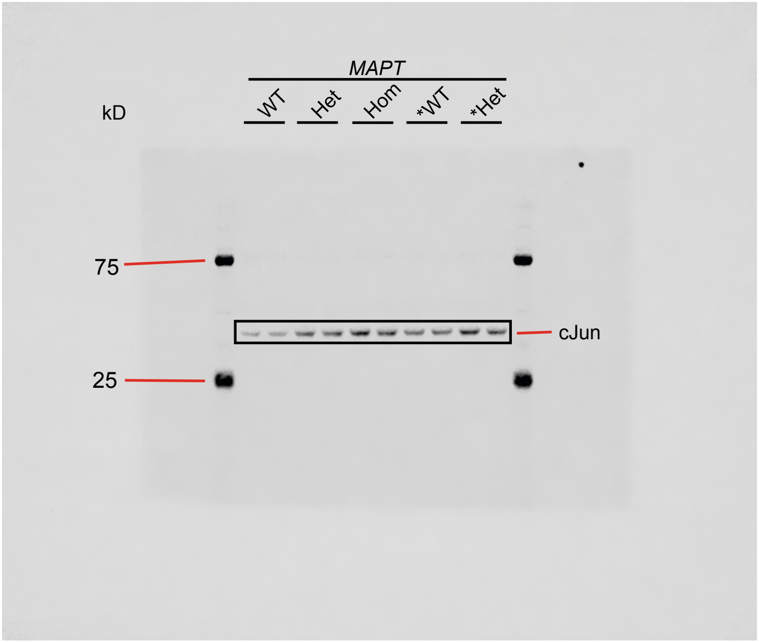

p-cJun - 800 channel

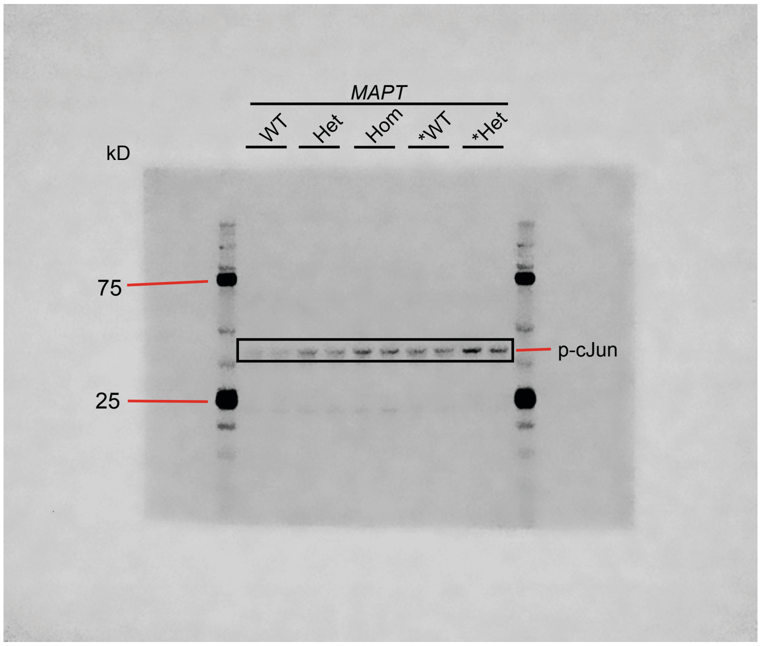

GAPDH - 700 channel

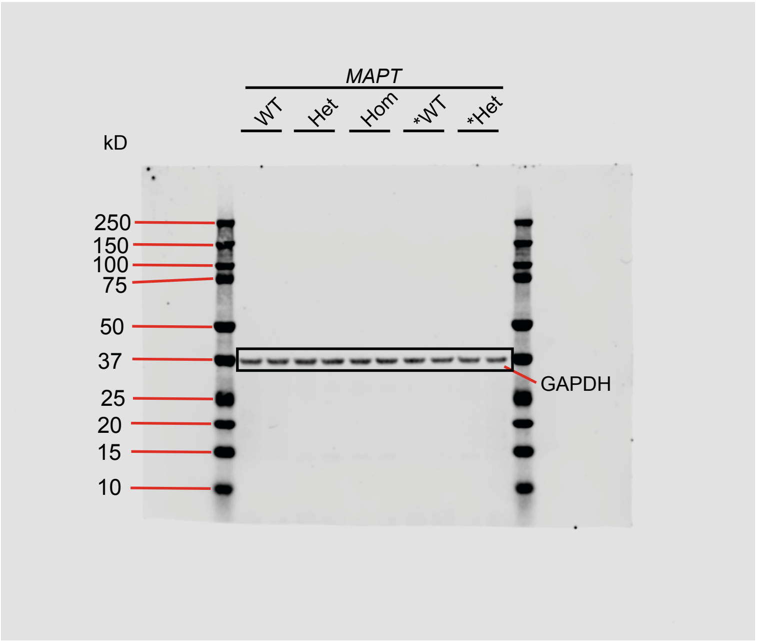

cJun - 800 channel

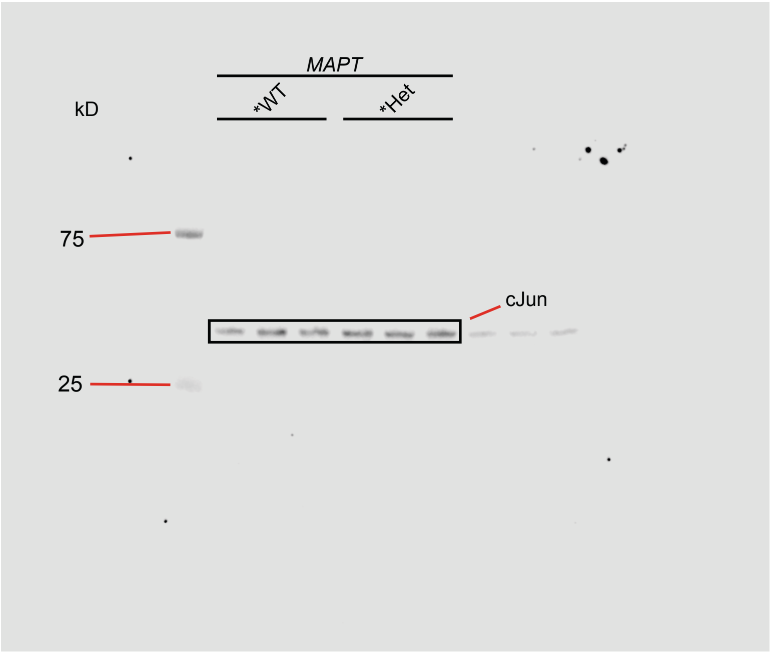

GAPDH - 700 channel

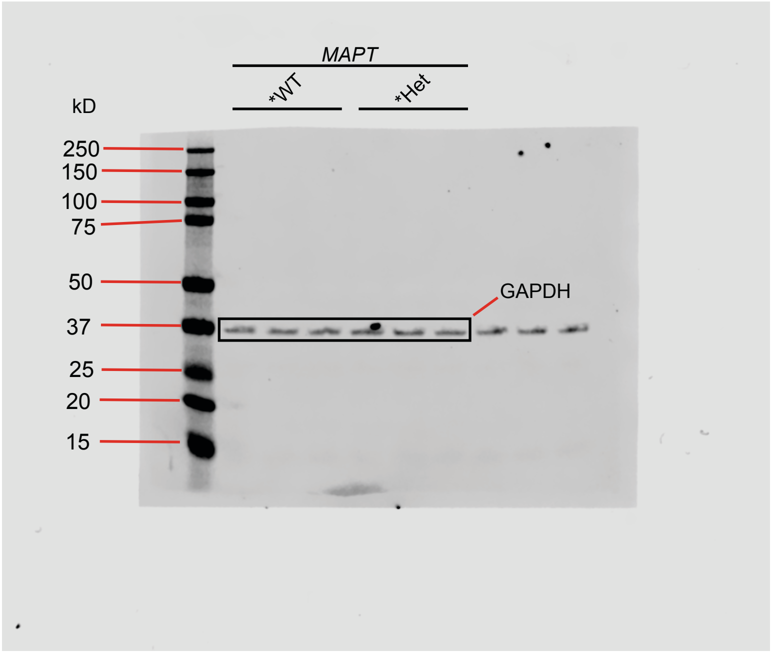

p-cJun - 800 channel

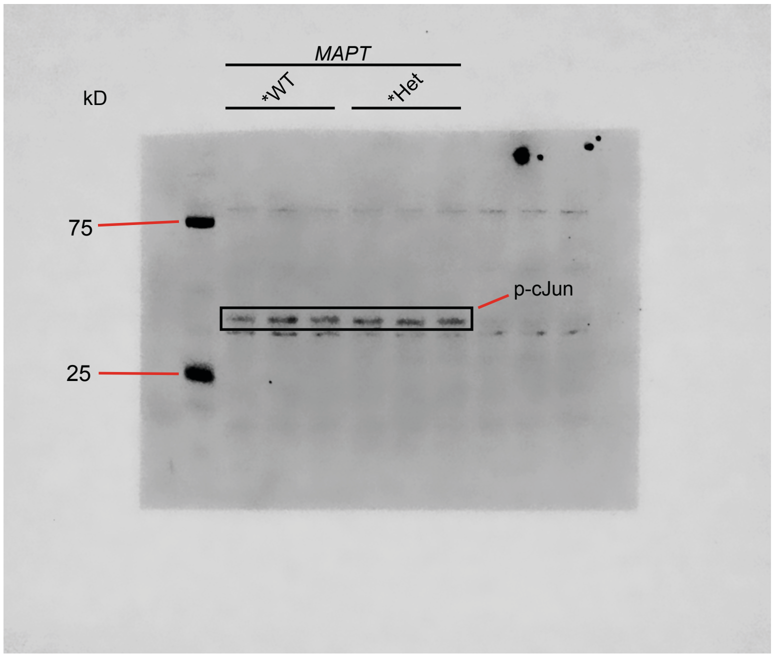

Source data for Fig. S2D

cJun - 800 channel

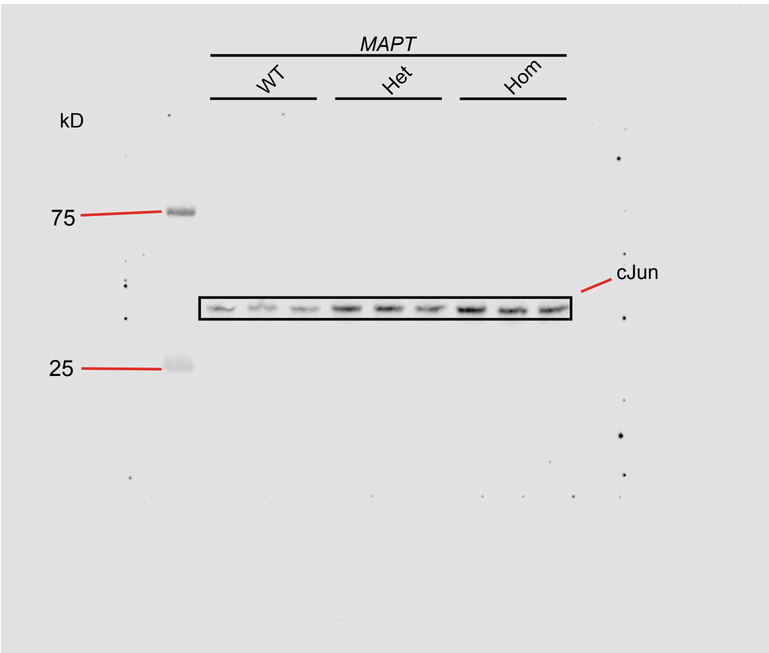

p-cJun - 800 channel

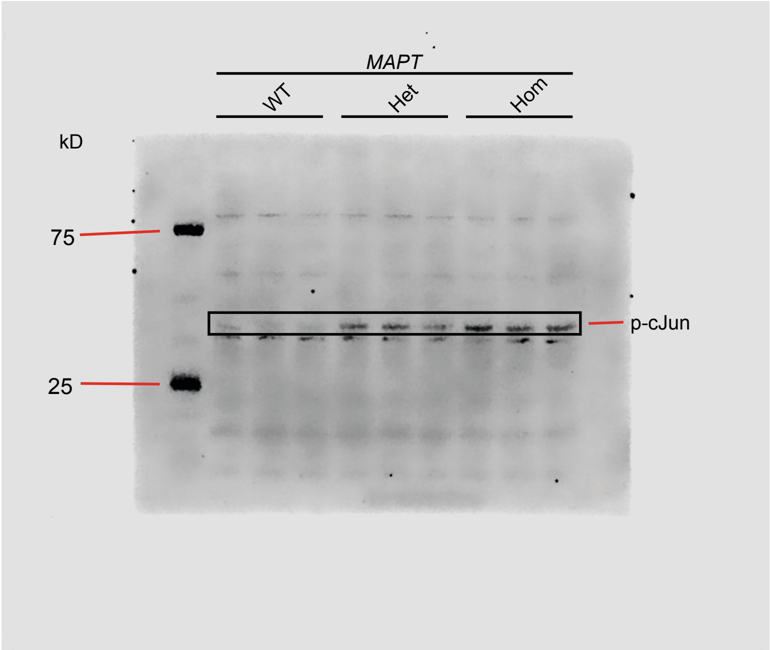

GAPDH - 700 channel

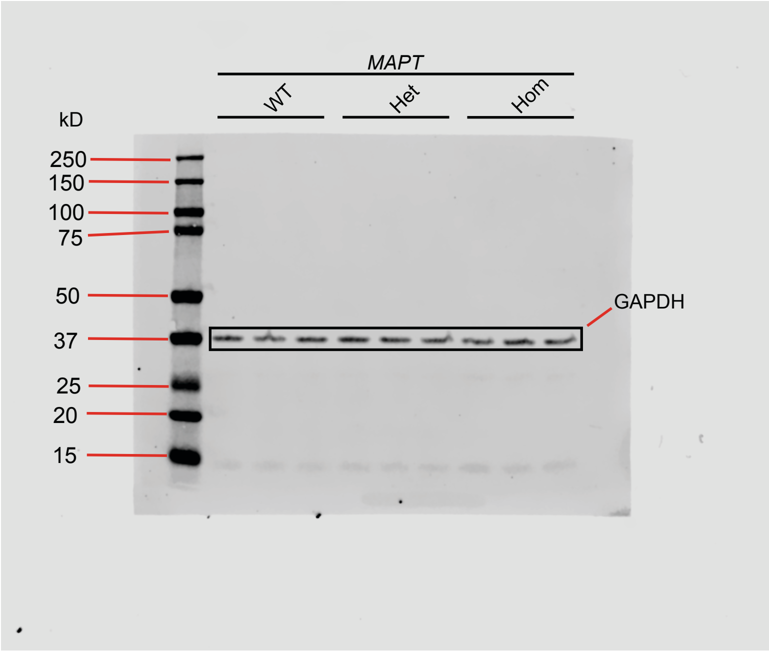

Source data for Fig. 4C

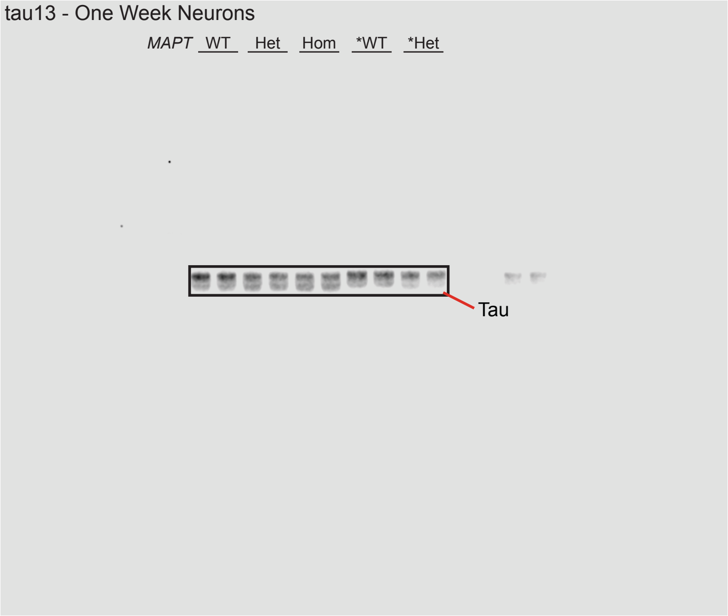

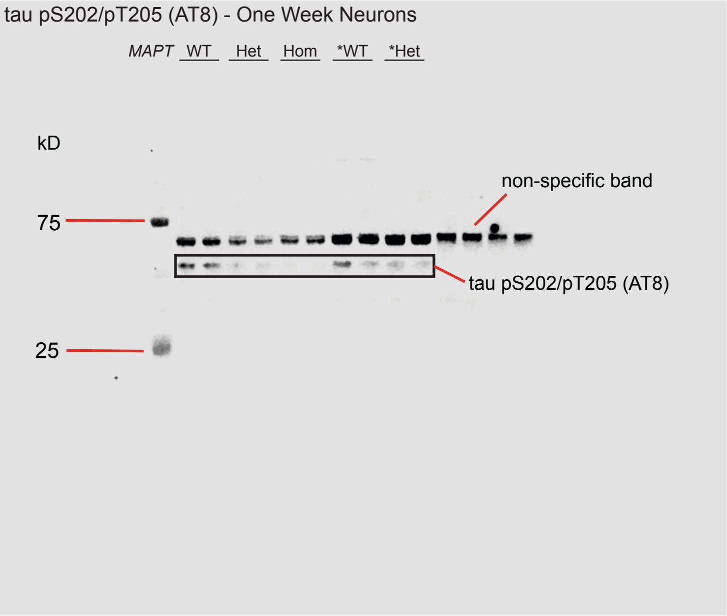

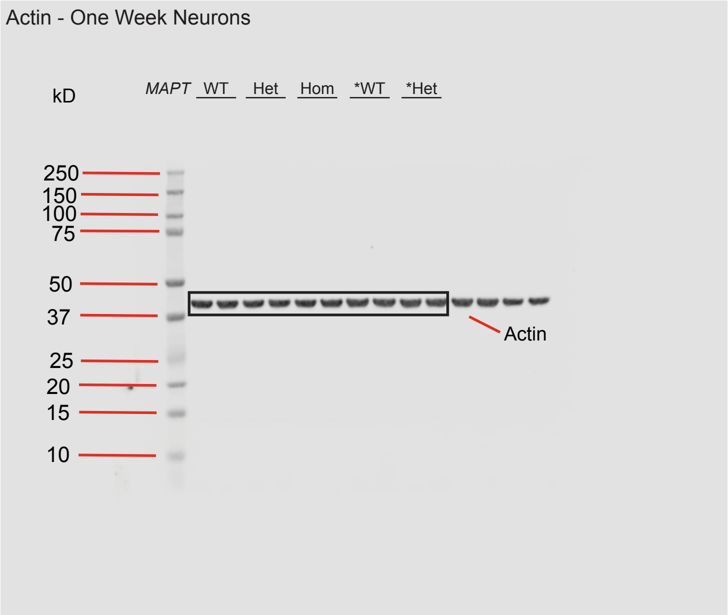

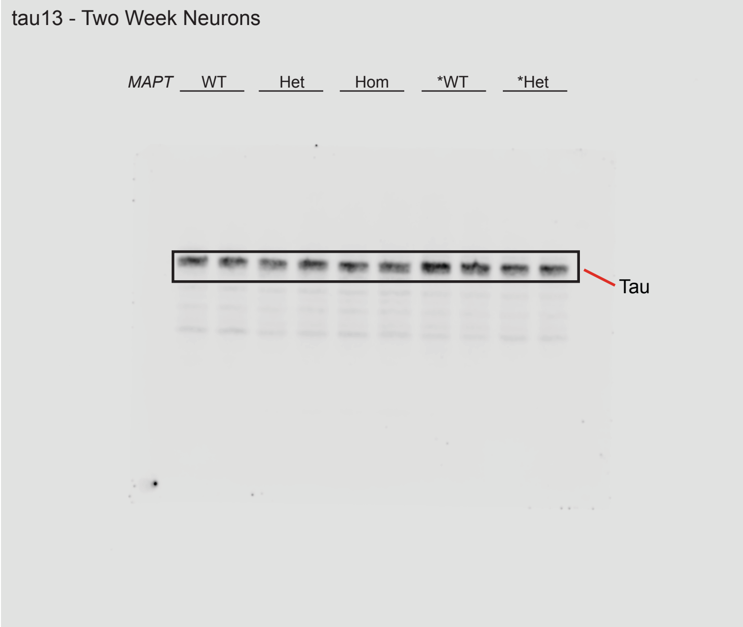

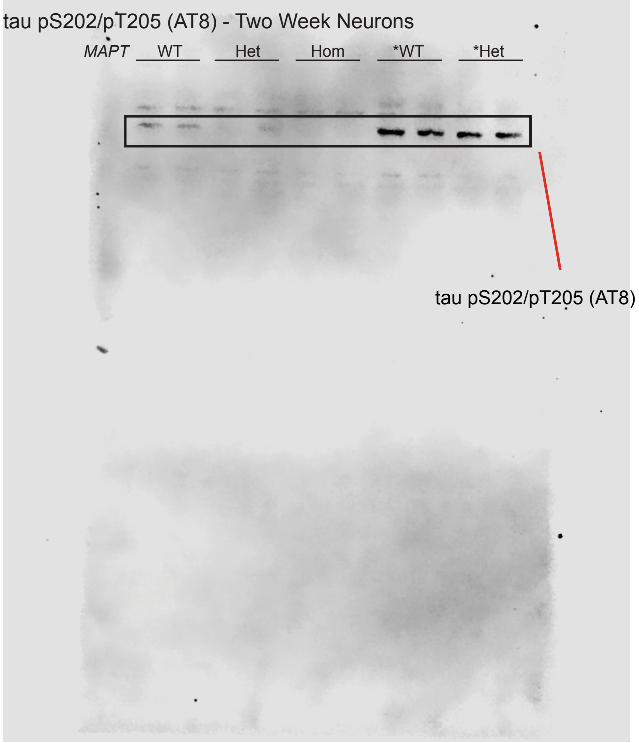

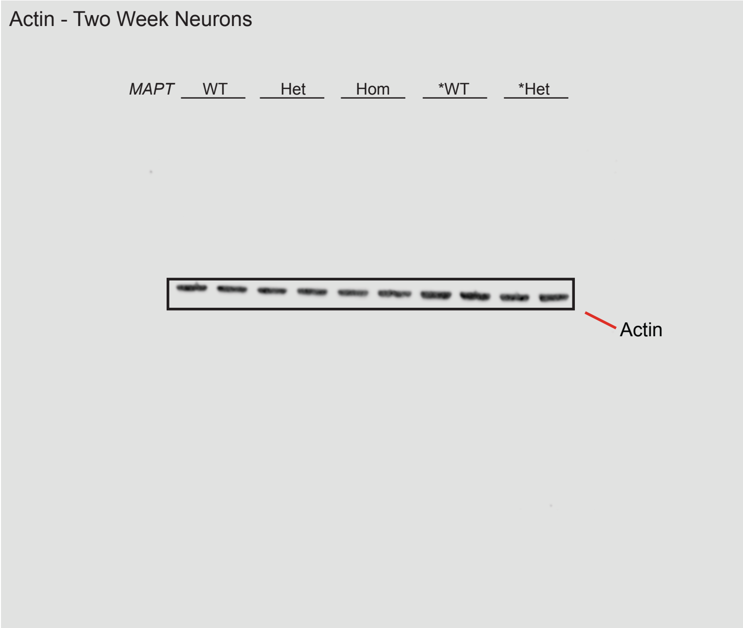

Source data for Fig. 4C

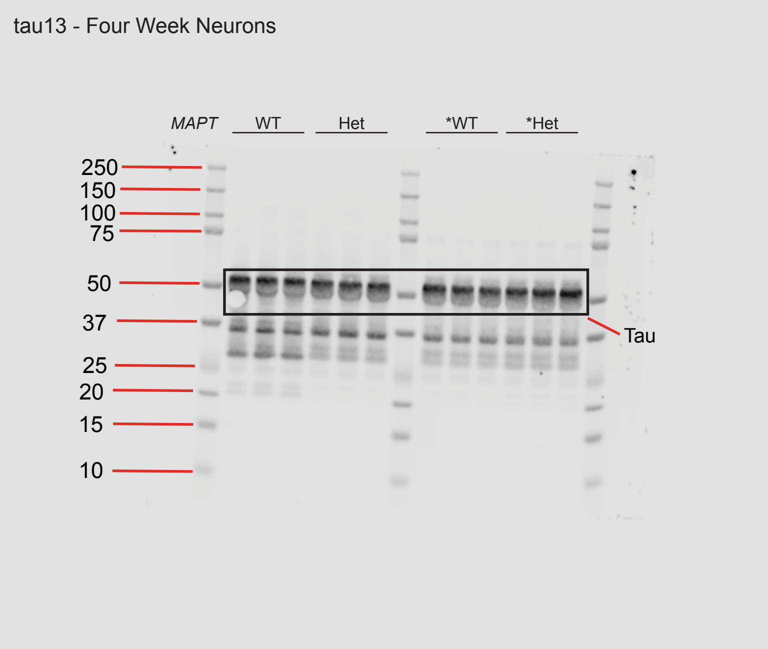

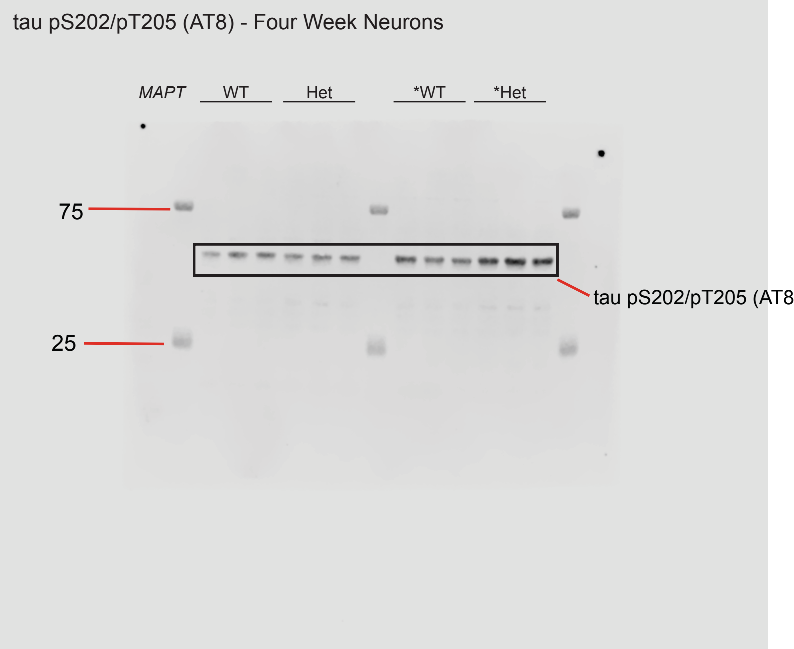

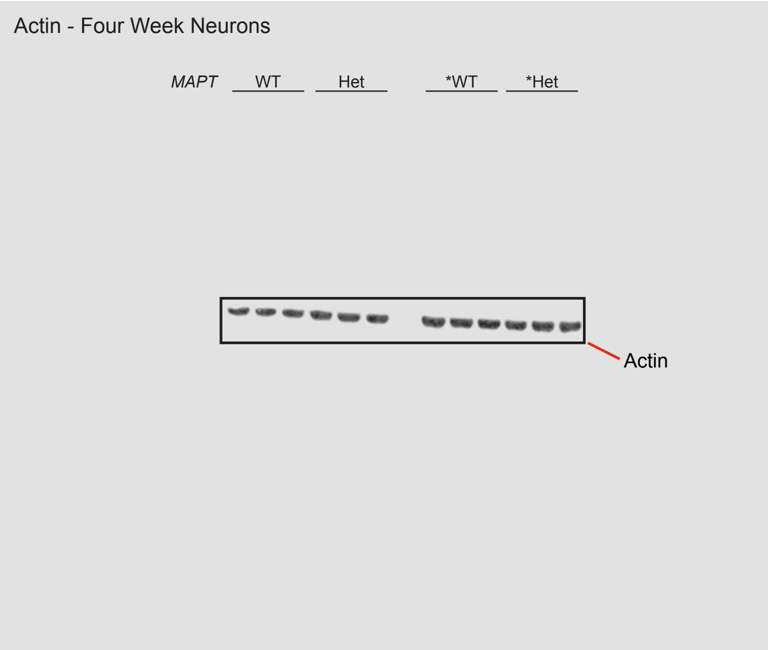

Source data for Fig. 4E

tau pS202/pT205 (AT8)

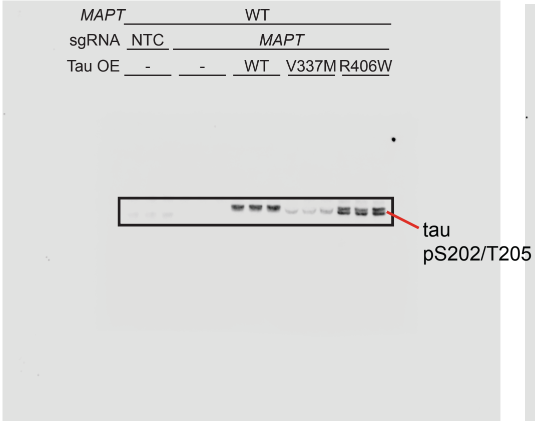

tau pT212/pS214 (AT100)

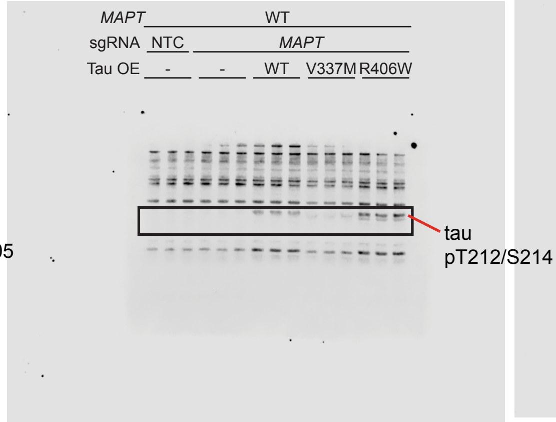

tau pT217

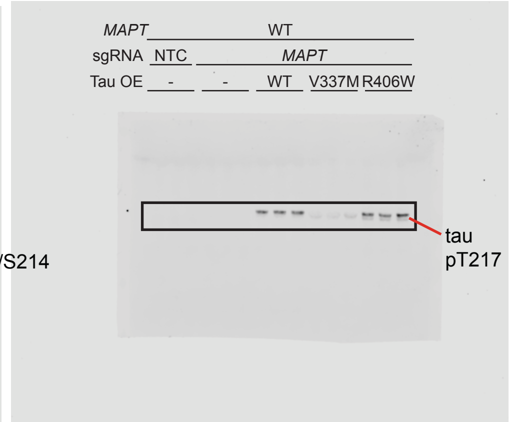

tau pT231 (AT180)

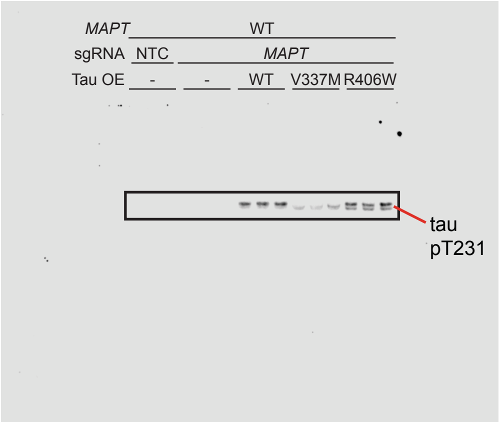

tau pS396

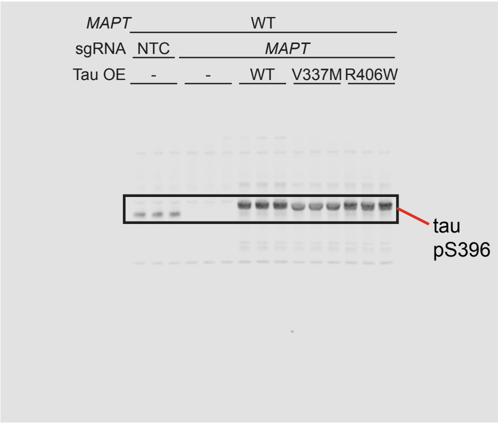

total tau

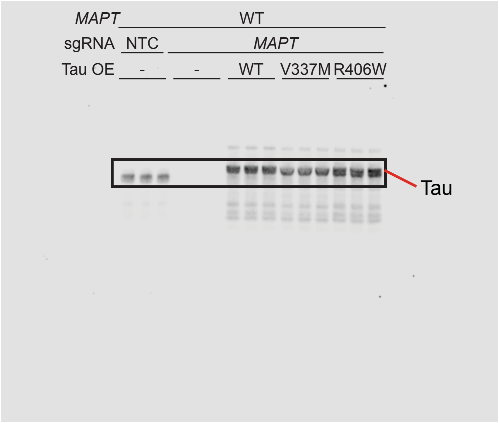

Actin

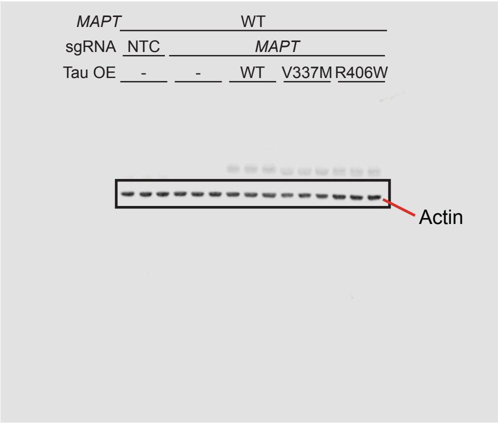

Source data for Fig. S6A

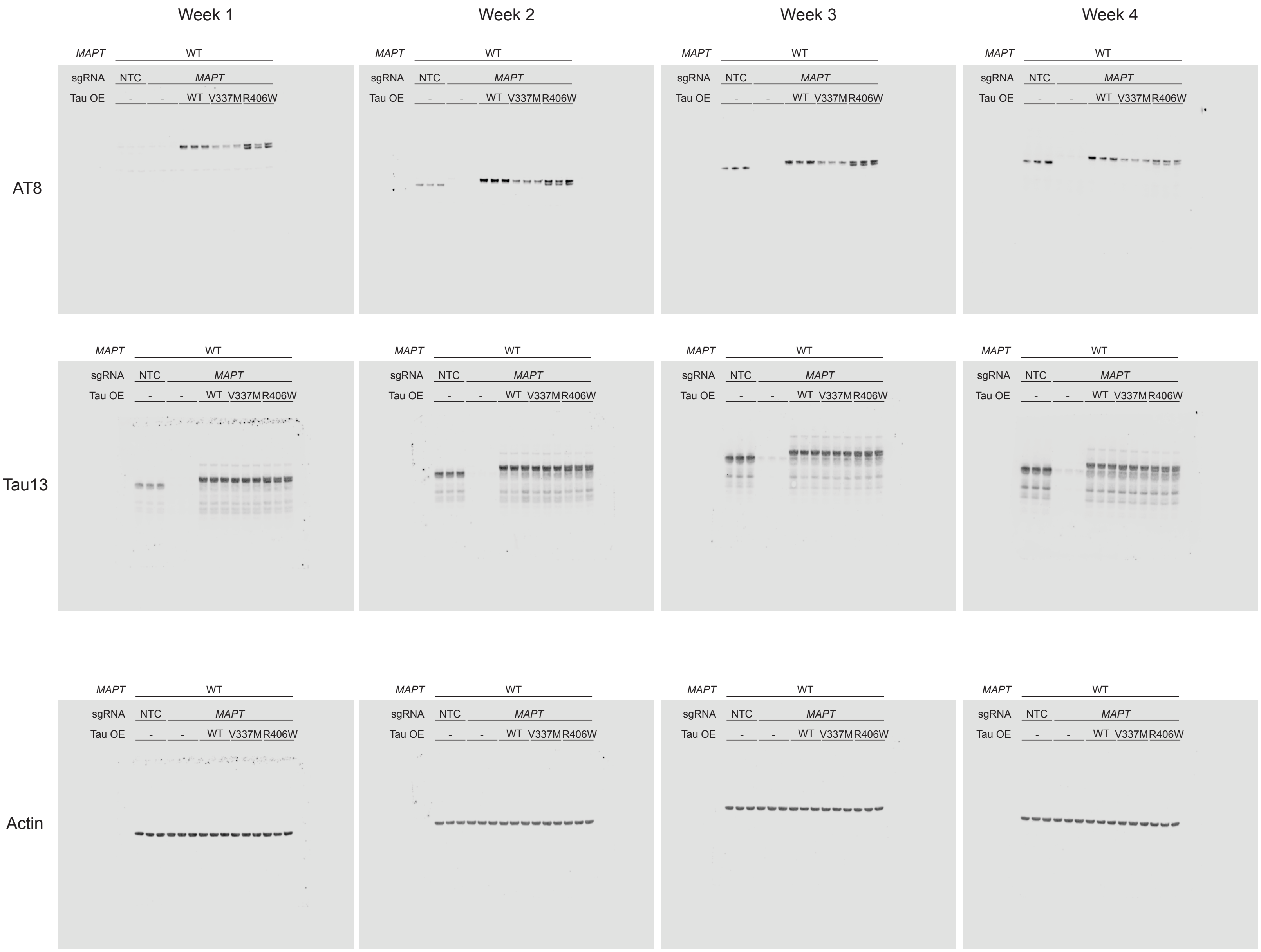

Source data for Fig. S6D

tau pS262

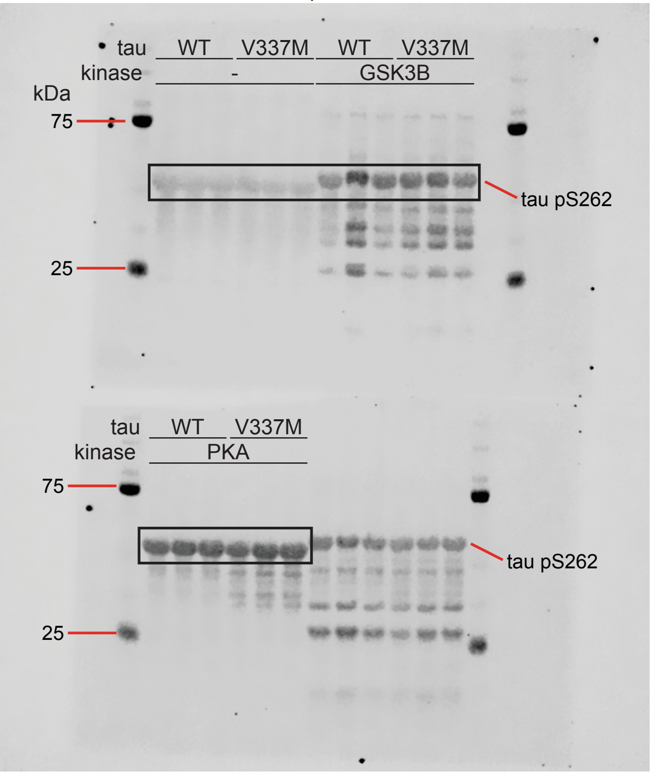

tau pS202/T205

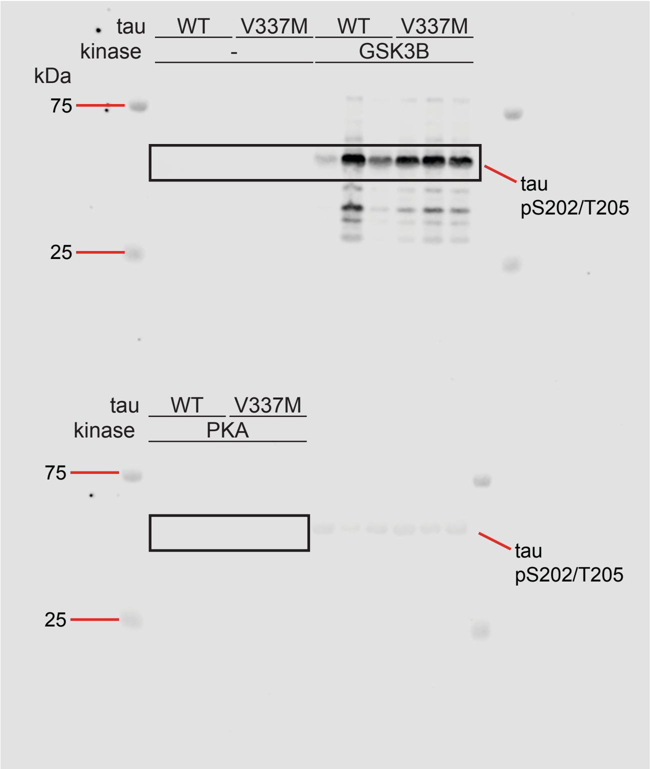

tau pT231

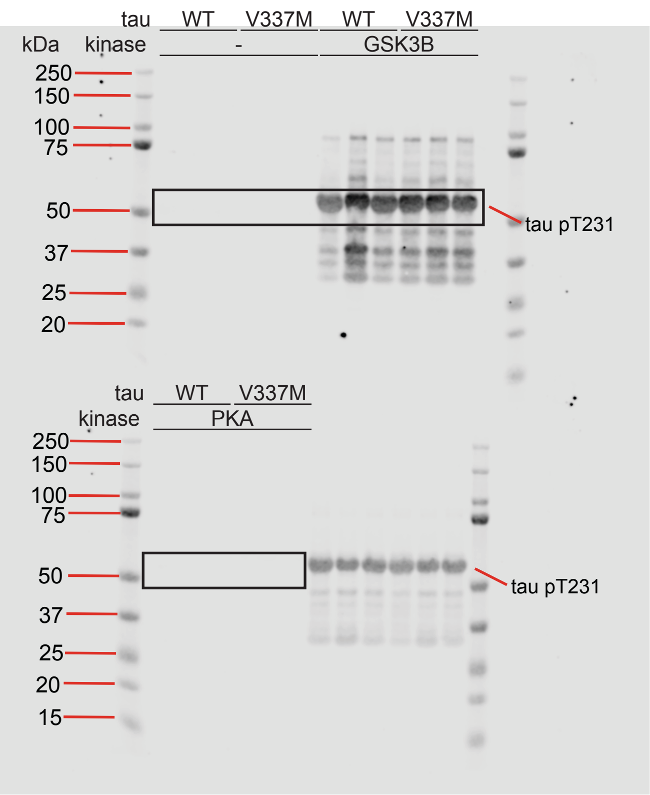

Sypro Ruby (Total Protein)

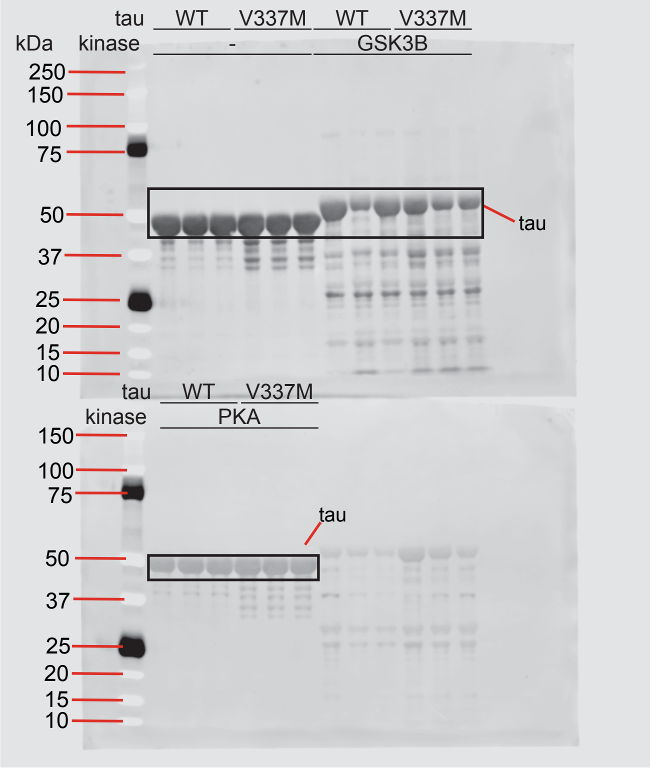

Supplementary Data

DataS1.xlsx	RNA-seq data
DataS2.xlsx	ATAC-seq data
DataS3.xlsx	Proteomics data
DataS4.xlsx	Predicted Kinase Activity
DataS5.xlsx	CRISPRi/a Screen Data

DataS6 Primers

Primer ID	Sequence
CC_Gib_0N3R/0N4R_Tau_fwd	TA TTA TTG AGG CTC ACA GAG AAC AGA TTG GTG GTA TGG CAG AGC CCC GCC AGG A
CC_Gib_0N3R/0N4R_Tau_rev	GGA TCC ATC GGT GCT ACC TGT TTG GTC TCA CAA ACC CTG CTT GGC CAG GGA G

DataS7.xlsx GO Gene Set Enrichment Analysis
